# Training-only ultrasound-specific augmentation for ovarian tumor segmentation across B-mode and contrast-enhanced ultrasound

**DOI:** 10.3389/fmed.2026.1878351

**Published:** 2026-07-13

**Authors:** Yini Wang, Luyu Hu, Zebin Xue, Qinzi Li, Jingze Li, Xiaoxia Kong

**Affiliations:** 1 Department of Gynecology, Guangdong Engineering Research Center of AI-Powered Precision Cancer Diagnostics and Therapeutics (Proposed)/GuangDong Engineering Technology Research Center of AI-Powered Precision Cancer Diagnostics and Therapeutics, Cancer Hospital of Shantou University Medical College, Shantou, China; 2 Department of Neurosurgery, The First Affiliated Hospital of Shantou University Medical College, Shantou, China; 3 College of Life Sciences, Sichuan Agricultural University, Ya'an, China; 4 College of Information Engineering, Sichuan Agricultural University, Ya'an, China

**Keywords:** contrast-enhanced ultrasound, data augmentation, domain shift, ovarian tumor, segmentation

## Abstract

**Background:**

Ovarian tumor segmentation models trained on conventional ultrasound may be sensitive to contrast-enhanced ultrasound appearance. We evaluated whether training-only combined ultrasound-specific augmentation could improve robustness without adding inference-time complexity.

**Methods:**

We used MMOTU image-mask pairs, including 820 development B-mode ultrasound images, 382 internal two-dimensional ultrasound images, and 170 contrast-enhanced ultrasound images as a CEUS domain-shift stress test. A lightweight Residual U-Net was compared with the same backbone trained using photometric, blur, and low-amplitude noise augmentation. Performance was evaluated using overlap, boundary, and pixel-calibration metrics. Paired CEUS differences were tested with Wilcoxon signed-rank tests, and effect sizes, 95% confidence intervals, and case-level failure rates were reported.

**Results:**

The combined augmentation preserved internal performance, with Dice of 0.745 (0.196) compared with 0.750 (0.191) for Residual U-Net. On contrast-enhanced ultrasound, it improved Dice from 0.468 (0.190) to 0.476 (0.192), intersection over union from 0.326 (0.167) to 0.334 (0.171), Boundary F1 from 0.037 (0.035) to 0.041 (0.032), and pixel expected calibration error from 0.314 (0.143) to 0.297 (0.138). The mean paired CEUS Dice improvement was 0.008 (95% CI -0.0002 to 0.0167); 62.9% of cases improved and 37.1% worsened. However, the proportion of CEUS cases with Dice below 0.5 decreased from 55.9 to 51.8%, indicating that CEUS segmentation remained suboptimal.

**Conclusion:**

Training-only combined ultrasound-specific augmentation yielded a small but directionally favorable CEUS improvement without added inference-time complexity. These findings support training-distribution design as a practical low-cost strategy for modality-shift robustness and warrant further patient-level and multi-center validation.

## 1 Introduction

Ovarian cancer remains a major cause of gynecologic cancer mortality, and the clinical pathway for adnexal masses depends on timely risk assessment, referral, and treatment planning (1–3). Ultrasound is central to this pathway because it is widely available, does not expose patients to ionizing radiation, and provides real-time morphologic assessment of ovarian and adnexal lesions. International efforts have therefore emphasized standardized terminology, structured ultrasound features, and risk-stratification systems for adnexal masses, including the International Ovarian Tumor Analysis framework, the ADNEX model, and O-RADS ultrasound (4–9). These systems demonstrate that imaging phenotypes are clinically meaningful, but they also depend on consistent visual interpretation and reproducible feature extraction.

Segmentation is not identical to diagnosis, yet it is a necessary technical step for many quantitative ultrasound workflows. Delineating a tumor or lesion region can support size measurement, shape analysis, radiomic feature extraction, longitudinal comparison, and downstream model development. In ovarian ultrasound, segmentation is challenging because lesion boundaries may be partly obscured by acoustic shadowing, heterogeneous echogenicity, posterior enhancement, and variable probe angle. Manual delineation is also time-consuming and can vary with experience, display settings, and the amount of contextual information available during image review. These constraints make reproducible automated segmentation attractive, but they also make it easy for a model to learn appearance patterns that are specific to one acquisition setting rather than the lesion boundary itself.

The problem becomes more complex when images come from different ultrasound modes. Contrast-enhanced ultrasound introduces vascular and perfusion-related intensity patterns that can alter local texture and boundary appearance, and clinical studies have evaluated CEUS as an adjunct for adnexal mass characterization (10–13). A segmentation model that performs acceptably on conventional two-dimensional ultrasound may therefore not transfer cleanly to CEUS, even when the anatomic target remains similar. This issue is methodologically different from ordinary random test error: it reflects a structured shift in image contrast, spatial detail, and background texture. A clinically useful segmentation study should therefore ask not only whether a model fits the development distribution, but also whether its probability maps and boundaries remain reliable when the acquisition domain changes.

Deep convolutional segmentation models, especially U-Net variants, have become standard tools in medical image analysis because they combine local detail with multiscale context in relatively data-efficient architectures (14–19). However, higher architectural complexity does not by itself solve domain shift. Medical imaging models may learn scanner-, institution-, protocol-, or modality-specific shortcuts, and performance can change substantially when the deployment distribution differs from the development distribution (20). For ultrasound, the difficulty is amplified by operator dependence, speckle, gain settings, acquisition depth, and postprocessing. These factors argue for methodologic studies that test robustness and calibration rather than reporting only within-domain overlap metrics.

Data augmentation is a natural strategy for limited-data medical segmentation because it can expose the model to plausible appearance variation without requiring additional labeled images (21). Recent medical image segmentation studies have investigated augmentation and domain-generalization methods for single-source training, style variation, and acquisition variability (22–25). In practice, however, augmentation can help, harm, or leave performance unchanged depending on whether the perturbations reflect clinically plausible variation. Rather than proposing a novel augmentation operator, this study evaluates whether a training-distribution strategy composed of commonly available perturbations—motivated by the specific appearance differences between B-mode and contrast-enhanced ultrasound—can systematically improve CEUS robustness without adding inference-time complexity. The contribution is therefore methodologic and evaluative rather than algorithmic.

Reliability also extends beyond Dice or intersection over union. A model can produce a similar average overlap while becoming less calibrated, less stable near lesion boundaries, or more heterogeneous across cases. Calibration is especially relevant for medical AI because a probability map is often interpreted as more than a binary mask, and poorly calibrated probabilities can undermine thresholding, visual review, and downstream quantitative use (26–30). For a public image-level ovarian ultrasound dataset without verified patient identifiers, this makes transparent data partitioning and clinically bounded claims especially important (31–35).

In this study, we evaluated a lightweight clinical-methodologic approach for ovarian tumor segmentation across two-dimensional ultrasound and CEUS. Rather than enlarging the backbone or adding inference-time modules, we trained a Residual U-Net with a combined ultrasound-specific augmentation profile combining photometric, blur, and low-amplitude noise perturbations. Using the public MMOTU image-mask resource, we defined a deterministic development split, a held-out internal two-dimensional ultrasound test set, and a CEUS domain-shift stress test. The objective was to determine whether training-only augmentation could improve CEUS overlap, boundary behavior, and pixel-level calibration while preserving internal two-dimensional ultrasound performance. We emphasize that the perturbations employed are individually well-known; the methodologic value lies in their systematic, ultrasound-motivated combination and in the simultaneous evaluation of overlap, boundary, calibration, and efficiency under a modality-shift stress test.

## 2 Methods

### 2.1 Study design and data source

**Figure 1:**
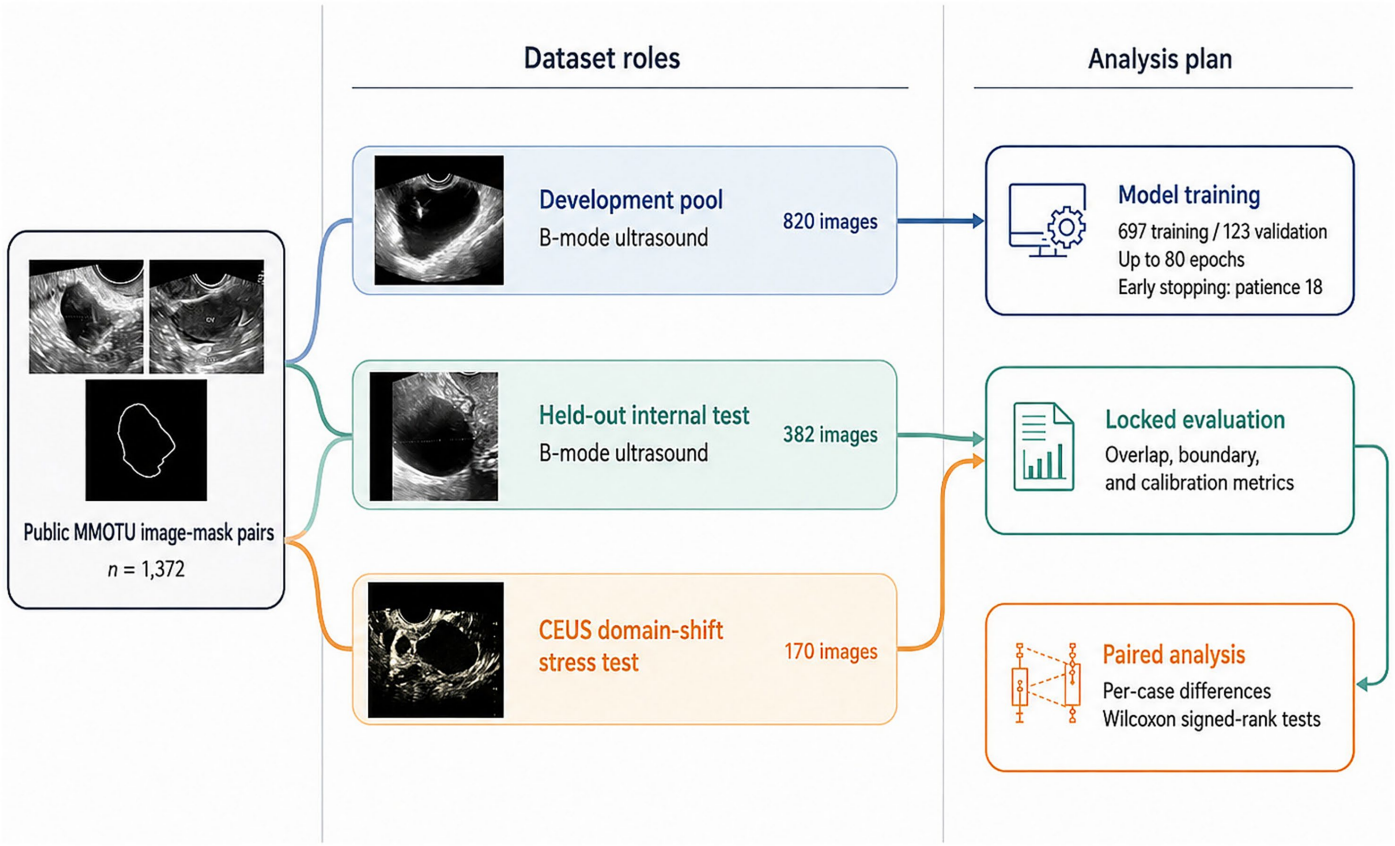
Study workflow and evaluation design.

This image-level methodologic study used public MMOTU ovarian tumor ultrasound image-mask pairs (36–38). The workflow is shown in Figure 1. The available local dataset comprised 820 two-dimensional ultrasound images assigned to the development pool, 382 held-out two-dimensional ultrasound images assigned to the internal test set, and 170 contrast-enhanced ultrasound images assigned to the CEUS domain-shift stress test. The development pool was split deterministically into 697 training images and 123 validation images using a hash-based split with seed 42. Table 1 summarizes the image-level dataset characteristics. Because verified patient identifiers were not available in the working public package, all analyses were conducted at the image level, and no patient-level baseline table or patient-level inference was constructed. Exact duplicates were assessed using MD5 hashes of raw pixel data. Near-duplicate images were screened using an 8 × 8 DCT-based perceptual hash with a Hamming-distance threshold of ≤5, with particular attention to matches crossing dataset partitions.

**Table 1:** Image-level dataset characteristics.

Modality	Dataset role	*N* images	Median width (px)	Median height (px)	Width range (px)	Height range (px)
2D ultrasound	Held-out internal 2D ultrasound test	382	955	538	407–1,135	266–794
2D ultrasound	Development pool; deterministic 697/123 train/validation split	820	956	538	319–1,135	270–794
Contrast-enhanced ultrasound	CEUS domain-shift stress test	170	553	552	330–888	218–657

Values summarize image-level records from the public dataset package.

The public dataset package provided paired image and mask files. Images were loaded as three-channel RGB arrays to maintain compatibility with common convolutional implementations, and masks were loaded as single-channel grayscale images. The segmentation foreground was defined as mask intensity greater than 127. Images and masks were resized to 512 × 512 pixels; bilinear interpolation was used for images, and nearest-neighbor interpolation was used for masks after thresholding. Image intensities were scaled to [0,1] and normalized using ImageNet channel means and standard deviations. This was used as a standardized preprocessing choice and was not intended to imply natural-image transfer learning.

The held-out internal test set was used to assess whether the method preserved performance on conventional two-dimensional ultrasound from the same dataset family. The CEUS set was used as a CEUS domain-shift stress test because CEUS changes the image appearance distribution while retaining the same organ-specific segmentation target. This terminology reflects the available image-level metadata, because institution-level identifiers and patient-level sampling were not available.

### 2.2 Segmentation backbone

The primary backbone was a lightweight Residual U-Net with three encoder levels, a bottleneck, bilinear decoder upsampling, skip concatenations, and a one-channel output layer. The base channel width was 32. Each convolutional block used two 3 × 3 convolutions with batch normalization and rectified linear activation. Residual connections were used in deeper encoder, bottleneck, and decoder blocks when input and output channels matched. The model used approximately 1.95 million trainable parameters. This backbone was selected to test whether training-distribution design could improve CEUS behavior without moving to a larger architecture.

For comparison, we evaluated a standard U-Net with the same base channel width and an Attention U-Net variant using attention-gated skip connections. The Attention U-Net comparator was included as a common architectural refinement baseline, not as a claim that attention mechanisms were optimized for this dataset. To assess whether the augmentation benefit was model-specific, all three backbones (U-Net, Residual U-Net, and Attention U-Net) were trained with both standard augmentation and the combined ultrasound-specific augmentation profile. All augmentation variants used the same Residual U-Net backbone for the primary ablation analysis, so differences among those variants reflect training-time image perturbation rather than model size.

### 2.3 Ultrasound-domain augmentation

**Figure 2:**
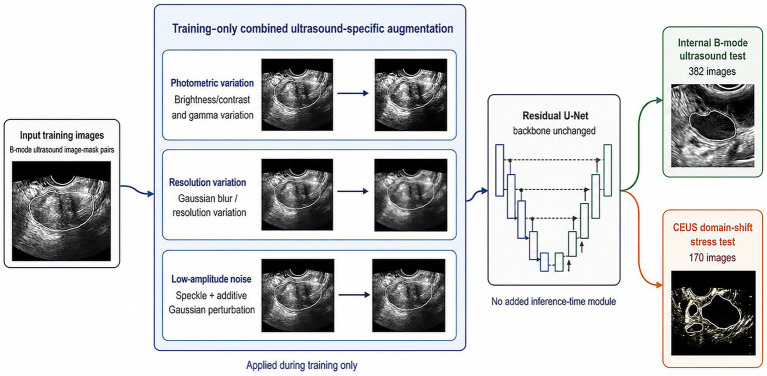
Training-only combined ultrasound-specific augmentation protocol. Brightness/contrast and gamma variation, Gaussian blur, and low-amplitude speckle and additive Gaussian perturbations were applied during training, while the Residual U-Net inference backbone remained unchanged.

The method contribution was a training-only combined ultrasound-specific augmentation profile, shown in Figure 2. The profile was designed to approximate plausible appearance variation in ovarian ultrasound while leaving the inference model unchanged. Standard augmentation, applied to all models including baselines, comprised random horizontal and vertical flips (probability 0.5 each) and brightness and contrast adjustment (probability 0.5; multiplicative factors sampled uniformly from 0.85 to 1.15). The combined profile then added three ultrasound-oriented perturbation families during training: photometric gamma variation, resolution or blur variation, and low-amplitude noise variation.

For photometric variation, a gamma transformation was applied with probability 0.5 using gamma sampled uniformly from 0.75 to 1.35. For resolution and blur variation, Gaussian blur was applied with probability 0.35 using radius sampled uniformly from 0.2 to 1.1 pixels. For low-amplitude noise variation, multiplicative speckle noise was applied with probability 0.55 using a standard deviation sampled uniformly from 0.02 to 0.08, and additive Gaussian noise was applied with probability 0.35 using a standard deviation sampled uniformly from 0.005 to 0.025. Pixel values were clipped to [0, 1] after array-level perturbation. The binary mask was transformed only by the same geometric operations as the image and was not altered by intensity perturbations.

The augmentation parameters were empirically prespecified as mild perturbation ranges based on ultrasound appearance variability and development-set validation, rather than derived from a formal fit to the CEUS test distribution. The gamma range (0.75–1.35) was chosen to cover a moderate brightness range that could plausibly arise from differences in ultrasound gain settings and imaging mode, without reference to the CEUS test distribution; the blur radius (0.2–1.1 pixels) approximated moderate resolution loss that can arise from differing probe frequencies and focal depths; and the low-amplitude noise (speckle standard deviation 0.02–0.08; additive standard deviation 0.005–0.025) was kept small to avoid overwhelming the weak lesion-boundary signal. Parameter selection did not use the CEUS test set for tuning. A summary of the augmentation operations, application probabilities, parameter ranges, and selection rationale is provided in Supplementary Table S1.

Let x denote the input image and y the binary mask. The full training transform can be written as


$$T_{\text{full}}(x,y)=T_{\mathrm{geo}}(T_{\text{noise}}(T_{\text{blur}}(T_{\text{gamma}}(x))),y)$$ (1)

where $T_{\mathrm{geo}}$ denotes shared image-mask flips and $T_{\text{gamma}}$ , $T_{\text{blur}}$ , and $T_{\text{noise}}$ denote image-only perturbations sampled from the distributions above (Equation 1). At inference, $T_{\text{full}}$ was not applied, and the model received only the standardized input image.

### 2.4 Training objective and optimization

For an image i with binary target mask $Y_{i}$ and model logits $z_{i}$ , the foreground probability map was $p_{i}=\text{sigmoid}\;(z_{i})$ . The primary objective combined binary cross-entropy with soft Dice loss:


$$L=\mathrm{BCE}(z_{i},Y_{i})+1-\frac{2\sum_{p}p_{i}Y_{i}+\in }{\sum_{p}p_{i}+\sum_{p}Y_{i}+\in }$$ (2)

where the sums are over image pixels and *ε* = 10^−6^ (Equation 2). We note that the smoothing constant is denoted ε throughout; the prime symbol appearing in the original equation rendering was a typographical artifact and has been corrected. This objective was used for the primary Residual U-Net, the combined augmentation profile, the component ablations, the standard U-Net, and the Attention U-Net comparator. Dice-based losses are commonly used in medical segmentation when foreground regions are relatively small or imbalanced (39, 40). Boundary- or calibration-weighted auxiliary losses were not used in the final primary method, because the manuscript question focused on training-distribution design rather than added loss terms.

Models were trained with AdamW, initial learning rate $10^{-3}$ , weight decay $10^{-4}$ , batch size 8, and cosine annealing over a maximum of 80 epochs. Dropout was set to 0.10 in the training configuration. Early stopping was applied when validation Dice did not improve for 18 epochs, and the checkpoint with the highest validation Dice was selected for final evaluation. Random seeds for Python, NumPy, and PyTorch were fixed at 42. Training and evaluation were conducted on a single NVIDIA RTX 4090 GPU with 24 GB memory using PyTorch 2.5.1, Python 3.12, and CUDA 12.4.

### 2.5 Baselines and ablations

The principal comparison was between Residual U-Net with standard augmentation and Residual U-Net with the combined ultrasound-specific augmentation profile. Three component ablations were used to evaluate whether the combined profile was explained by a single perturbation family: photometric augmentation only, noise augmentation only, and blur augmentation only. The standard U-Net and Attention U-Net provided architecture comparators. To assess training stability, five-fold cross-validation was performed on the 820-image development pool: for each fold, the model was trained on four folds and validated on the held fold, then evaluated on the fixed internal and CEUS test sets. Because patient identifiers were unavailable, this cross-validation was image-level and cannot guarantee patient-level independence across folds. The ablation analysis was interpreted as methodologic evidence about training-distribution design, not as a search for the largest single metric improvement.

### 2.6 Evaluation metrics

Performance was assessed using complementary overlap and boundary metrics alongside pixel-level calibration, consistent with recommendations to use task-appropriate metric sets for medical image-analysis validation (41). Binary predictions were obtained by thresholding probability maps at 0.5. Overlap was assessed using Dice and intersection over union. For a predicted binary mask P and reference mask Y , Dice was defined as (Equation 3).


$$\begin{array}{c} \text{Dice}(P,Y)=\frac{2|P\cap Y|}{|P|+|Y|} \end{array}$$ (3)

and intersection over union was defined as (Equation 4).


$$\begin{array}{c} \mathrm{IoU}(P,Y)=\frac{|P\cap Y|}{|P\cup Y|} \end{array}$$ (4)

Pixel calibration was assessed using expected calibration error with 10 bins. For each pixel, confidence was defined as p for predicted foreground pixels and 1−p for predicted background pixels. Correctness was defined by agreement between the thresholded prediction and the reference mask. If $B_{b}$ denotes the set of pixels in bin b , the pixel expected calibration error was (Equation 5).


$$\begin{array}{c} \mathrm{ECE}=\sum_{b}\frac{|B_{b}|}{N}|\text{accuracy}\;(B_{b})-\text{confidence}\;(B_{b})| \end{array}$$ (5)

where N is the number of evaluated pixels. Lower pixel ECE indicates better agreement between predicted confidence and observed pixel correctness. Calibration was treated as a secondary reliability measure rather than as a direct clinical outcome.

Boundary accuracy was assessed using Boundary F1 and the 95th percentile Hausdorff distance (HD95). Boundary F1 was computed using a two-pixel tolerance around binary mask edges: precision was the fraction of predicted edge pixels within the tolerance of the reference edge, and recall was the fraction of reference edge pixels within the tolerance of the predicted edge. When both predicted and reference masks were empty, Boundary F1 was defined as 1.0; when either was empty, it was defined as 0.0. HD95 was calculated as the 95th percentile of the pooled bidirectional boundary distance distribution between predicted and reference mask edges, in image-pixel units. When both masks were empty, HD95 was defined as 0; when either was empty, HD95 was set to a large value (image diagonal). Lower HD95 values indicate smaller boundary outlier distances. Because all images were resized to 512 × 512 pixels before evaluation, HD95 values are reported in resized-pixel units and should not be interpreted as physical millimeter distances.

### 2.7 Statistical analysis

Performance values are reported as mean (standard deviation) across images. Paired augmentation effects on the CEUS domain-shift test were calculated as candidate minus Residual U-Net for each image (Equation 6):


$$\begin{array}{c} \Delta_{m,i}=m_{\text{candidate},i}-m_{\text{reference},i} \end{array}$$ (6)

For Dice, intersection over union, and Boundary F1, positive differences are favorable. For HD95 and pixel ECE, negative differences are favorable. Wilcoxon signed-rank tests were used for paired CEUS comparisons because the analysis was conducted on matched images. *p* values were interpreted descriptively to support the methodologic comparison; no patient-level clinical hypothesis was tested. In addition to mean differences and *p*-values, we reported median paired differences, 95% bootstrap confidence intervals (5,000 resamples), the proportion of cases that improved versus worsened, and case-level failure rates (proportion of images with Dice below 0.1, 0.2, and 0.5) to convey practical significance beyond statistical significance. Efficiency was summarized using parameter count, inference time per image, peak GPU memory during fixed inference measurement, and training time. The bootstrap confidence interval estimated the magnitude of the mean paired difference, whereas the Wilcoxon signed-rank test evaluated the distribution of paired differences.

### 2.8 Quantitative characterization of the B-mode–CEUS domain shift

To characterize the image-distribution differences between B-mode ultrasound (BUS) and CEUS, seven image-level statistics were computed on the original grayscale images (converted from RGB using standard luminance weighting) before resizing: mean intensity (0–255 scale), intensity standard deviation, dynamic range (99th percentile minus 1st percentile), root-mean-square contrast, Shannon entropy (256-bin histogram, in bits), Laplacian sharpness variance (variance of the Laplacian filter response, as a measure of high-frequency energy), and lesion area fraction (proportion of pixels with mask intensity above 127). Image borders, text annotations, and machine interface elements were not separately masked, as the public dataset did not provide region-of-interest metadata. Statistics were computed per image and summarized as mean and standard deviation within each modality group. Because this analysis was intended as a descriptive characterization rather than a prespecified hypothesis test, no formal inferential comparison was performed.

## 3 Results

### 3.1 Image-level dataset and evaluation roles

The final evaluation used 1,372 public image-mask pairs divided into a development pool, a held-out internal two-dimensional ultrasound test set, and a CEUS domain-shift stress test. Table 1 shows that the development pool contained 820 two-dimensional ultrasound images, with a deterministic 697/123 training-validation split. The internal test set contained 382 two-dimensional ultrasound images, and the CEUS stress test contained 170 contrast-enhanced ultrasound images. The median image dimensions differed by modality, with the two-dimensional ultrasound subsets having median widths around 955 to 956 pixels and the CEUS subset having a median width of 553 pixels and median height of 552 pixels. These image-level differences support the planned use of CEUS as a distribution-shift assessment rather than as an interchangeable extension of the internal two-dimensional test set.

Figure 1 places these dataset roles within the study workflow. The figure emphasizes that model development, internal testing, and CEUS stress testing were separated before performance analysis. Figure 2 then defines the training-only augmentation strategy used by the primary method. Together, these Methods figures establish the central comparison evaluated in the Results: whether changing the training image distribution improves CEUS behavior while leaving the Residual U-Net inference architecture unchanged.

### 3.2 Primary segmentation performance

**Figure 3:**
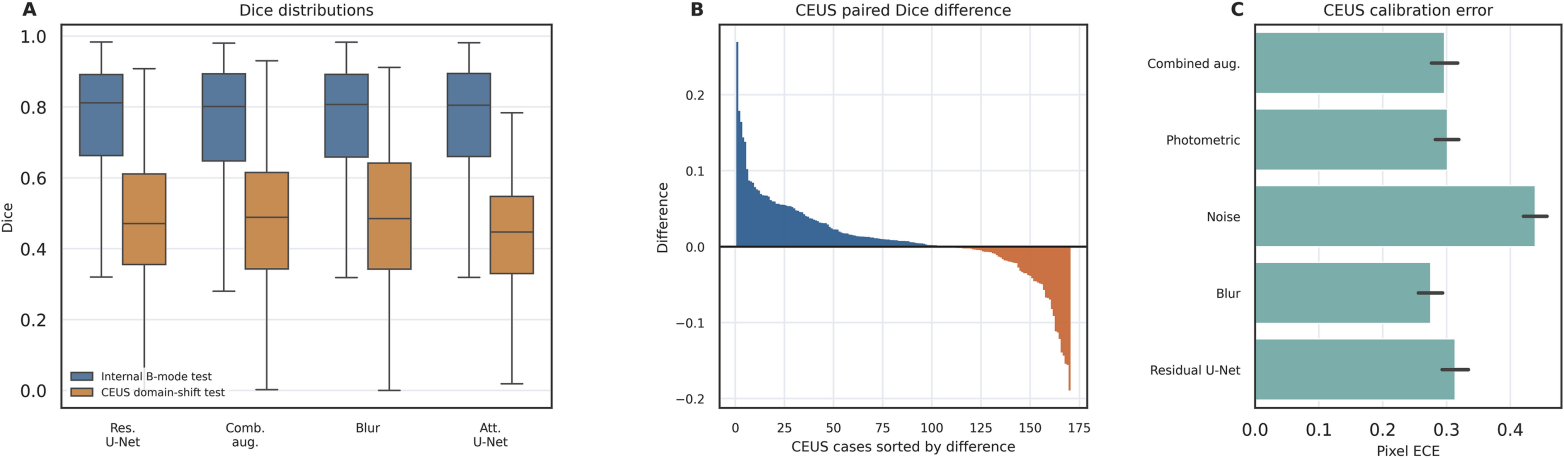
Primary segmentation performance. **(A)** Dice distributions for Residual U-Net, combined augmentation, blur augmentation, and Attention U-Net on the internal B-mode and CEUS domain-shift tests. **(B)** Per-case CEUS Dice differences between combined augmentation and Residual U-Net. **(C)** Pixel expected calibration error on the CEUS domain-shift test; bars show means with 95% confidence intervals.

Table 2 and Figure 3 summarize the primary segmentation results. On the held-out internal two-dimensional ultrasound test set, the Residual U-Net achieved Dice of 0.750 (0.191), IoU of 0.632 (0.216), Boundary F1 of 0.167 (0.123), HD95 of 97.986 (50.627), and pixel ECE of 0.039 (0.044). The combined ultrasound-specific augmentation profile produced similar internal overlap, with Dice of 0.745 (0.196) and IoU of 0.628 (0.221), while Boundary F1 increased to 0.178 (0.133), HD95 decreased to 93.448 (50.716), and pixel ECE decreased to 0.037 (0.042). This pattern indicates that the combined augmentation did not materially trade away internal two-dimensional ultrasound performance for the CEUS comparison.

**Table 2:** Segmentation performance on internal 2D ultrasound and CEUS domain-shift tests.

Model	Evaluation set	N images	Dice	IoU	Boundary F1	HD95	Pixel ECE
Residual U-Net	Internal 2D ultrasound test	382	0.750 (0.191)	0.632 (0.216)	0.167 (0.123)	97.986 (50.627)	0.039 (0.044)
Residual U-Net	CEUS domain-shift test	170	0.468 (0.190)	0.326 (0.167)	0.037 (0.035)	199.683 (65.159)	0.314 (0.143)
Combined augmentation	Internal 2D ultrasound test	382	0.745 (0.196)	0.628 (0.221)	0.178 (0.133)	93.448 (50.716)	0.037 (0.042)
Combined augmentation	CEUS domain-shift test	170	0.476 (0.192)	0.334 (0.171)	0.041 (0.032)	197.557 (64.357)	0.297 (0.138)
Blur augmentation	Internal 2D ultrasound test	382	0.748 (0.196)	0.631 (0.220)	0.168 (0.126)	99.917 (52.583)	0.037 (0.042)
Blur augmentation	CEUS domain-shift test	170	0.477 (0.212)	0.338 (0.187)	0.050 (0.037)	203.073 (64.607)	0.275 (0.132)
Photometric augmentation	Internal 2D ultrasound test	382	0.752 (0.190)	0.635 (0.217)	0.173 (0.127)	96.156 (49.826)	0.038 (0.043)
Photometric augmentation	CEUS domain-shift test	170	0.465 (0.190)	0.323 (0.165)	0.043 (0.033)	201.750 (64.956)	0.302 (0.124)
Noise augmentation	Internal 2D ultrasound test	382	0.746 (0.193)	0.627 (0.217)	0.167 (0.118)	98.379 (51.834)	0.039 (0.045)
Noise augmentation	CEUS domain-shift test	170	0.429 (0.158)	0.287 (0.136)	0.017 (0.019)	208.041 (68.450)	0.439 (0.126)
Attention U-Net	Internal 2D ultrasound test	382	0.749 (0.190)	0.631 (0.217)	0.168 (0.129)	98.487 (53.943)	0.038 (0.042)
Attention U-Net	CEUS domain-shift test	170	0.429 (0.175)	0.289 (0.146)	0.035 (0.028)	204.505 (65.856)	0.328 (0.132)
U-Net	Internal 2D ultrasound test	382	0.752 (0.189)	0.635 (0.214)	0.166 (0.123)	98.914 (51.314)	0.039 (0.044)
U-Net	CEUS domain-shift test	170	0.439 (0.162)	0.296 (0.141)	0.024 (0.028)	202.188 (66.009)	0.374 (0.114)

Values are mean (SD).

On the CEUS domain-shift stress test, all methods had lower performance than on the internal two-dimensional ultrasound test, consistent with a substantial modality shift. The Residual U-Net had CEUS Dice of 0.468 (0.190), IoU of 0.326 (0.167), Boundary F1 of 0.037 (0.035), HD95 of 199.683 (65.159), and pixel ECE of 0.314 (0.143). The combined augmentation improved CEUS Dice to 0.476 (0.192), IoU to 0.334 (0.171), Boundary F1 to 0.041 (0.032), HD95 to 197.557 (64.357), and pixel ECE to 0.297 (0.138). Although the absolute overlap gains were small, the paired changes were directionally consistent across overlap, boundary, and calibration measures.

To convey practical significance, the mean paired CEUS Dice improvement was 0.008 (median 0.007; 95% CI -0.0002 to 0.0167), with 62.9% of cases improving and 37.1% worsening. The 95% confidence interval for Dice included zero, indicating that the improvement, while directionally favorable and statistically significant (*p* < 0.001), was modest in magnitude. Case-level failure rates underscored the suboptimal CEUS performance: 55.9% of CEUS cases had Dice below 0.5 for the baseline and 51.8% for the combined augmentation, while 8.2 and 8.2% had Dice below 0.2, respectively. No cases produced empty predictions (Dice = 0). These results indicate that statistical significance does not imply clinical significance, and that current CEUS performance remains limited.

Figure 3A shows that CEUS Dice distributions remained lower than internal two-dimensional ultrasound distributions for all selected methods. This is important for interpretation: the combined augmentation reduced the CEUS gap but did not remove the domain shift. Figure 3B shows paired CEUS Dice differences between the combined augmentation and Residual U-Net, demonstrating heterogeneous case-level effects with both improved and worsened cases. Figure 3C shows the calibration pattern on CEUS, where the combined augmentation had lower pixel ECE than Residual U-Net and lower pixel ECE than the Attention U-Net comparator.

The architecture comparators did not explain the main CEUS improvement. Attention U-Net achieved internal Dice of 0.749 (0.190), close to Residual U-Net, but CEUS Dice decreased to 0.429 (0.175), and CEUS pixel ECE was 0.328 (0.132). The standard U-Net achieved internal Dice of 0.752 (0.189), but CEUS Dice was 0.439 (0.162), with CEUS pixel ECE of 0.374 (0.114). These results support the interpretation that the observed CEUS improvement was more consistent with training-domain design than with adding a larger or attention-gated comparator.

### 3.3 Qualitative CEUS examples

**Figure 4:**
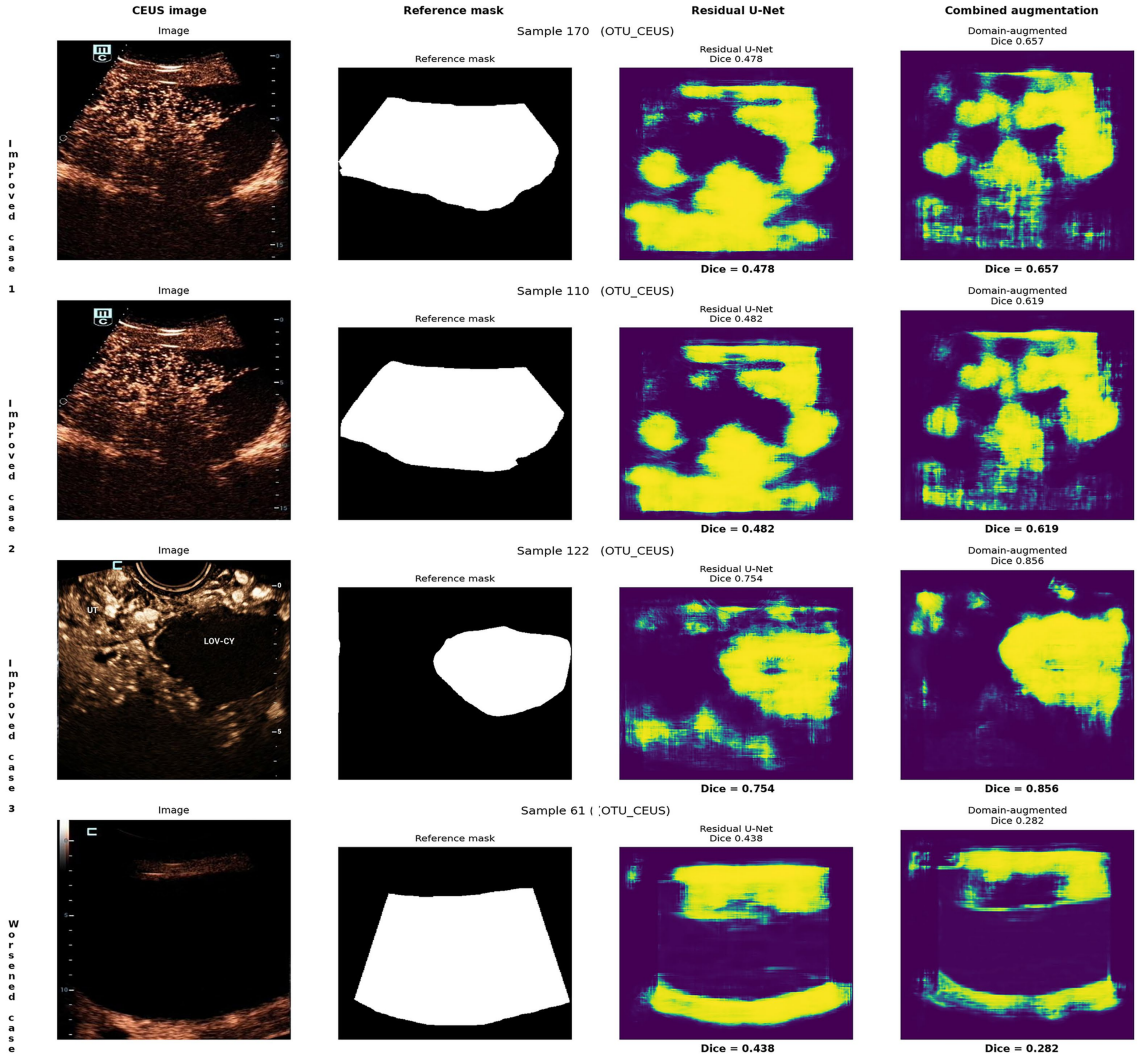
Representative CEUS segmentation examples illustrating heterogeneous responses to combined ultrasound-specific augmentation. Each row shows the CEUS image, reference mask, Residual U-Net probability map, and combined-augmentation probability map with the corresponding Dice score. The first three rows show improved Dice, whereas the final row shows lower Dice after augmentation.

Figure 4 provides representative CEUS examples illustrating heterogeneous case-level responses to combined ultrasound-specific augmentation. The first three rows show improved Dice relative to the Residual U-Net baseline, whereas the final row shows lower Dice after augmentation. These examples complement the paired quantitative findings by demonstrating that the average favorable shift was not uniform across individual cases.

### 3.4 Augmentation-component analysis

**Figure 5:**
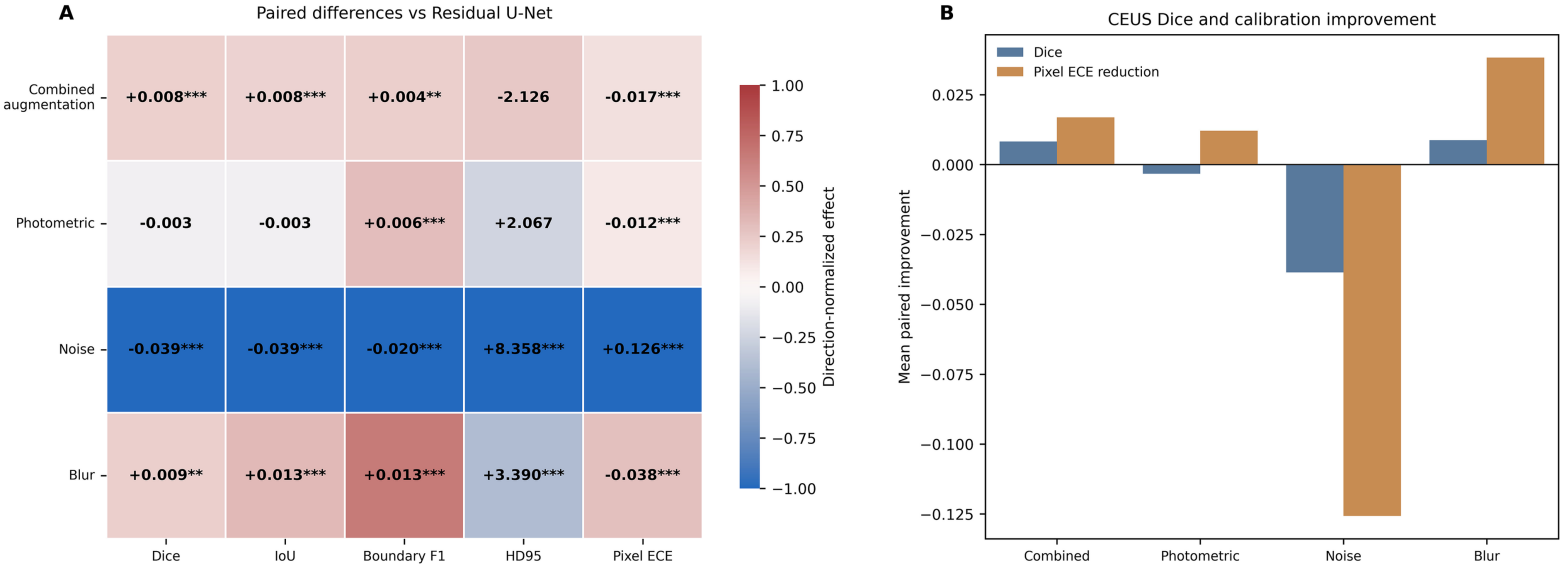
Augmentation-component analysis on the CEUS domain-shift test. **(A)** Direction-normalized paired differences relative to Residual U-Net, with raw mean differences annotated in each cell. Asterisks denote two-sided Wilcoxon signed-rank test significance: *p < 0.05, **p < 0.01, and ***p < 0.001. **(B)** CEUS Dice improvement and pixel ECE reduction relative to Residual U-Net; positive values indicate improvement.

Table 3 and Figure 5 evaluate whether a single augmentation component explained the CEUS improvement. Relative to Residual U-Net, the combined augmentation improved CEUS Dice by 0.008 (median 0.007; 95% CI −0.0002 to 0.0167; *p* < 0.001), IoU by 0.008 (*p* < 0.001), Boundary F1 by 0.004 (*p* = 0.005), and pixel ECE by −0.017 (*p* < 0.001). HD95 changed by −2.126 pixels (*p* = 0.158) and was not statistically significant. This profile was therefore balanced across overlap, boundary, and calibration measures, though the HD95 benefit was negligible.

**Table 3:** Paired augmentation ablation analysis on the CEUS domain-shift test.

Metric	Combined augmentation	Photometric augmentation	Noise augmentation	Blur augmentation
Dice	Diff. = 0.008; *p* < 0.001	Diff. = − 0.003; *p* = 0.353	Diff. = − 0.039; *p* < 0.001	Diff. = 0.009; *p* = 0.003
IoU	Diff. = 0.008; *p* < 0.001	Diff. = − 0.003; *p* = 0.336	Diff. = − 0.039; *p* < 0.001	Diff. = 0.013; *p* < 0.001
Boundary F1	Diff. = 0.004; *p* = 0.005	Diff. = 0.006; *p* < 0.001	Diff. = − 0.020; *p* < 0.001	Diff. = 0.013; *p* < 0.001
HD95	Diff. = − 2.126; *p* = 0.158	Diff. = 2.067; *p* = 0.081	Diff. = 8.358; *p* < 0.001	Diff. = 3.390; *p* < 0.001
Pixel ECE	Diff. = − 0.017; *p* < 0.001	Diff. = − 0.012; *p* < 0.001	Diff. = 0.126; *p* < 0.001	Diff. = − 0.038; *p* < 0.001

Paired differences are relative to Residual U-Net. *p* values were calculated using two-sided Wilcoxon signed-rank tests. For HD95 and Pixel ECE, lower values are favorable. Mean differences, 95% bootstrap confidence intervals, and the proportion of cases improved are reported in the supplement.

Photometric augmentation alone did not improve CEUS Dice or IoU, with Dice difference of −0.003 (*p* = 0.353) and IoU difference of −0.003 (*p* = 0.336), but it improved Boundary F1 by 0.006 (*p* < 0.001) and pixel ECE by −0.012 (*p* < 0.001). Noise augmentation alone was unfavorable across all CEUS measures, reducing Dice by −0.039 (*p* < 0.001), reducing Boundary F1 by −0.020 (*p* < 0.001), increasing HD95 by 8.358 pixels (*p* < 0.001), and increasing pixel ECE by 0.126 (*p* < 0.001). Blur augmentation improved Dice by 0.009 (*p* = 0.003), IoU by 0.013 (*p* < 0.001), Boundary F1 by 0.013 (*p* < 0.001), and pixel ECE by −0.038 (*p* < 0.001), but increased HD95 by 3.390 pixels (*p* < 0.001).

Figure 5A uses direction-normalized colors to show favorable and unfavorable directions across metrics while retaining raw paired mean differences in each cell. This visualization highlights the main methodologic finding: plausible acquisition-resolution variation was useful, arbitrary noise perturbation was harmful, and the combined augmentation provided the most balanced evidence across the prespecified CEUS metrics. Figure 5B focuses on Dice improvement and pixel ECE reduction, showing why the combined augmentation was selected as the primary method despite the strong calibration effect of blur-only augmentation. The combined augmentation avoided the significant HD95 penalty seen with blur alone while retaining CEUS overlap and calibration gains.

Exploratory heterogeneity analysis examined whether the augmentation-induced CEUS Dice difference was associated with baseline segmentation difficulty. The Spearman correlation between baseline Dice and the paired Dice difference was *ρ* = 0.064 (*p* = 0.410), indicating no significant monotonic relationship. Stratified by baseline difficulty, mid-difficulty cases (baseline Dice 0.3–0.6) showed the largest mean improvement (0.012; 67.0% improved), while high-baseline cases (≥0.6) showed negligible change (−0.001) and low-baseline cases (<0.3) improved modestly (0.011; 54.8% improved). This exploratory pattern suggests that the augmentation benefit, when present, was concentrated in cases of intermediate difficulty, but the absence of scanner, device, pathology, and acquisition metadata limits further mechanistic interpretation.

### 3.5 Efficiency and compute

Table 4 summarizes practical efficiency. The Residual U-Net and all augmentation variants had the same parameter scale, with 1.95 million parameters. The combined augmentation had inference time of 2.579 ms per image and peak GPU memory of 397.4 MB, compared with 2.595 ms per image and 396.6 MB for Residual U-Net. Training time was 20.4 min for the combined augmentation and 21.5 min for Residual U-Net under the measured configuration. Attention U-Net had a slightly larger parameter count of 1.98 million, inference time of 3.020 ms per image, and peak GPU memory of 455.2 MB.

**Table 4:** Model efficiency comparison.

Model	Parameters, million	Inference time (ms/image)	Peak GPU memory (MB)	Training time (min)
Residual U-Net	1.95	2.595	396.6	21.5
Combined augmentation	1.95	2.579	397.4	20.4
Blur augmentation	1.95	2.596	397.3	19.7
Photometric augmentation	1.95	2.605	397.6	19.9
Noise augmentation	1.95	2.612	397.6	19.7
Attention U-Net	1.98	3.020	455.2	21.4
U-Net	1.95	2.620	397.3	20.8

All augmentation variants use the Residual U-Net backbone; augmentation changes training only.

These efficiency results are central to the clinical-methodologic interpretation. The combined augmentation changed only training-time exposure to ultrasound-domain variation and did not add an inference-time module, uncertainty ensemble, or larger backbone. Supplementary Figure S2 provides a visual summary of these efficiency findings, but the main text relies on Table 4 because the practical claim is statistical and reproducibility-oriented rather than visual.

### 3.6 BUS-to-CEUS domain-shift characterization

Quantitative image-distribution analysis confirmed substantial differences between B-mode ultrasound (BUS, *n* = 1,202) and contrast-enhanced ultrasound (CEUS, *n* = 170). CEUS images had lower mean intensity (32.6 vs. 58.5), lower Shannon entropy (5.66 vs. 6.53 bits), but higher Laplacian sharpness (857 vs. 671), indicating darker, less textured but locally sharper images with more high-frequency content. The dynamic range was narrower for CEUS (186 vs. 203), and the lesion area fraction was slightly larger (0.21 vs. 0.17). These image-distribution differences are visualized in Supplementary Figure S1. These descriptive differences provide *post hoc* imaging context for the relevance of photometric, resolution-oriented, and low-amplitude noise perturbations; they were not used to tune the augmentation ranges. The domain shift is therefore not merely a brightness change but involves coordinated changes in intensity, texture, and sharpness, consistent with the perfusion-related appearance of CEUS.

### 3.7 Five-fold cross-validation

Table 5 summarizes the five-fold cross-validation results. Across the five folds, the combined augmentation showed a positive but heterogeneous CEUS effect. On the CEUS domain-shift test, standard augmentation achieved Dice of 0.454 ± 0.016 and HD95 of 205.1 ± 3.7 pixels, while combined augmentation achieved Dice of 0.463 ± 0.016 and HD95 of 200.6 ± 8.5 pixels. The mean paired CEUS Dice difference across folds was +0.009 ± 0.013 with 3 of 5 folds showing clear improvement, 1 essentially unchanged, and 1 showing a negligible decrease. On the internal BUS test, combined augmentation produced a small Dice decrease (−0.004 ± 0.002), confirming a modest internal-CEUS trade-off. CEUS HD95 improved by −4.4 ± 5.5 pixels on average, though with substantial fold-to-fold variability. This image-level cross-validation cannot guarantee patient-level independence because patient identifiers are unavailable; duplicate image checking (MD5 hash for exact duplicates; 8 × 8 DCT-based perceptual hash with Hamming distance ≤ 5 for near-duplicates) found 0 exact duplicates, 0 cross-subset matches, and 144 within-modality near-duplicates, none of which crossed the development and evaluation partitions. These results indicate that the average augmentation effect was positive across folds, although the magnitude and direction varied across individual folds. The five-fold CV CEUS Dice values (standard 0.454, combined 0.463) are slightly lower than the single-split results (standard 0.468, combined 0.476) because each fold used 656 training images, compared with 697 training images in the original deterministic split. The paired augmentation effect, however, is in the same direction as the single-split analysis on average.

**Table 5:** Five-fold cross-validation results (mean (SD) across folds).

Augmentation	Evaluation set	Dice	IoU	Boundary F1	HD95 (px)
Standard	Internal BUS test	0.756 (0.003)	0.642 (0.003)	0.176 (0.003)	98.0 (1.3)
Standard	CEUS domain-shift test	0.454 (0.016)	0.313 (0.015)	0.039 (0.007)	205.1 (3.7)
Combined	Internal BUS test	0.752 (0.003)	0.637 (0.003)	0.175 (0.003)	97.6 (0.8)
Combined	CEUS domain-shift test	0.463 (0.016)	0.324 (0.016)	0.045 (0.004)	200.6 (8.5)

### 3.8 Multi-model augmentation generalization

Table 6 summarizes the multi-backbone augmentation comparison. Positive mean CEUS Dice differences were observed across all three backbone architectures. On the CEUS domain-shift test, U-Net improved from Dice 0.439 (standard augmentation) to 0.469 (combined augmentation; mean difference +0.030), Attention U-Net improved from 0.429 to 0.477 (+0.048), and Residual U-Net improved from 0.468 to 0.476 (+0.008). Pixel ECE also improved for all three backbones: U-Net from 0.374 to 0.273, Attention U-Net from 0.328 to 0.257, and Residual U-Net from 0.314 to 0.297. The multi-backbone analysis was treated as a secondary descriptive comparison. Mean CEUS Dice differences were positive across all three evaluated backbones, although their magnitudes varied. All CEUS Dice values remained below 0.48, confirming that the augmentation provides a limited robustness benefit rather than solving the domain shift. Internal BUS performance was broadly preserved across the evaluated backbones. Standard augmentation baselines are from the original single-split training runs (R002, R003, R014); combined augmentation results for U-Net and Attention U-Net are from newly trained single-split runs (R021, R022).

**Table 6:** Multi-model augmentation comparison on the CEUS domain-shift test.

Backbone	Augmentation	CEUS Dice	CEUS IoU	CEUS Boundary F1	CEUS HD95 (px)	CEUS Pixel ECE
U-Net	Standard	0.439 (0.162)	0.296 (0.141)	0.024 (0.028)	202.2 (66.0)	0.374 (0.114)
U-Net	Combined	0.469 (0.201)	0.328 (0.170)	0.051 (0.032)	199.4 (63.4)	0.273 (0.135)
Residual U-Net	Standard	0.468 (0.190)	0.326 (0.167)	0.037 (0.035)	199.7 (65.2)	0.314 (0.143)
Residual U-Net	Combined	0.476 (0.192)	0.334 (0.171)	0.041 (0.032)	197.6 (64.4)	0.297 (0.138)
Attention U-Net	Standard	0.429 (0.175)	0.289 (0.146)	0.035 (0.028)	204.5 (65.9)	0.328 (0.132)
Attention U-Net	Combined	0.477 (0.177)	0.331 (0.156)	0.038 (0.022)	197.0 (62.9)	0.257 (0.136)

### 3.9 Training-only augmentation versus test-time augmentation

Table 7 summarizes the comparison of no TTA, two-view TTA, and four-view TTA on the internal BUS and CEUS domain-shift tests. On the CEUS domain-shift test, the combined-augmentation Residual U-Net used for TTA evaluation (RTTA) achieved Dice 0.458, pixel ECE 0.297, and inference time 2.73 ms without TTA. Two-view flip TTA improved Dice marginally to 0.459 and pixel ECE to 0.285 at 5.47 ms (2.0 × cost), while four-view flip TTA improved Dice to 0.474 and pixel ECE to 0.264 at 10.56 ms (3.9 × cost). HD95 was similar across all TTA modes (195.2–195.5 pixels), indicating that TTA primarily improved overlap and calibration rather than boundary outliers. On the internal BUS test, four-view TTA improved Dice from 0.755 to 0.760 but at 3.9 × inference cost. Within the same RTTA checkpoint, four-view TTA improved CEUS Dice from 0.458 to 0.474 but required 3.9-fold longer inference time. In contrast, training-only augmentation adds no inference-time module or additional forward pass. These results demonstrate that training-only augmentation preserves inference-time efficiency while TTA scales inference cost linearly with the number of views.

**Table 7:** Test-time augmentation comparison on internal BUS and CEUS domain-shift tests.

Evaluation set	TTA mode	Dice	HD95 (px)	Pixel ECE	Inference time (ms)	Peak GPU memory (MB)
Internal BUS	No TTA	0.755	96.7	0.035	2.73	374.0
Internal BUS	2View	0.757	94.1	0.032	5.48	379.0
Internal BUS	4View	0.760	91.2	0.030	10.56	380.9
CEUS domain-shift	No TTA	0.458	195.3	0.297	2.73	374.0
CEUS domain-shift	2View	0.459	195.2	0.285	5.47	379.0
CEUS domain-shift	4View	0.474	195.5	0.264	10.56	380.9

## 4 Discussion

This study evaluated a training-only combined ultrasound-specific augmentation strategy for ovarian tumor segmentation across conventional two-dimensional ultrasound and CEUS. The primary finding was that a training-only, clinically motivated augmentation profile yielded a small but directionally favorable improvement in CEUS overlap, boundary behavior, and pixel calibration while preserving internal two-dimensional ultrasound performance. The method did not depend on a larger architecture or an added inference-time reliability module. This makes the result computationally interpretable: the performance change arose from how the model was exposed to plausible ultrasound appearance variation during training, not from increasing model capacity. However, the absolute gains were modest, CEUS segmentation remained suboptimal, and the results should be interpreted as a modest methodologic contribution that requires further validation.

The clinical motivation for this design follows from the role of ultrasound in adnexal mass assessment. IOTA terminology, IOTA predictive models, ADNEX, and O-RADS have shown that structured ultrasound features can support risk stratification (4–9). Yet segmentation models are often evaluated as if image appearance were stable across acquisition modes. CEUS is not merely an intensity-shifted version of conventional ultrasound; it can emphasize perfusion, vascularity, and contrast-related texture patterns that change the visual cues used by a segmentation network (10–13). Treating CEUS as a CEUS domain-shift stress test therefore provides a meaningful way to ask whether a model trained on conventional ultrasound can maintain useful mask behavior when the appearance distribution changes.

The results also illustrate why average overlap alone is insufficient for medical AI segmentation. The combined augmentation improved CEUS Dice from 0.468 to 0.476 and IoU from 0.326 to 0.334. These absolute improvements are small, and the 95% confidence interval for the paired Dice difference included zero (95% CI − 0.0002 to 0.0167), underscoring that statistical significance does not equate to clinical significance. Over half of CEUS cases (55.9% at baseline) had Dice below 0.5, and 37.1% of cases worsened after augmentation, confirming that the benefit was heterogeneous and that current performance remains limited and requires further validation before clinical deployment. The calibration result is relevant because a segmentation probability map is often used visually or computationally before it is reduced to a binary mask. A lower pixel ECE suggests better agreement between predicted confidence and pixel correctness, which is a useful property for downstream review and threshold-dependent workflows (26–30). At the same time, the CEUS Dice distribution remained substantially below internal two-dimensional ultrasound performance, so the study should be interpreted as a limited robustness improvement under domain shift rather than as a solved cross-modality segmentation problem.

The ablation analysis provides the main methodologic insight. Blur augmentation alone improved several CEUS measures and reduced pixel ECE more than the combined augmentation, but it also worsened HD95. In segmentation, this matters because HD95 is sensitive to boundary outliers and large local deviations, which may affect measurement reliability even when average overlap improves. Noise-only augmentation was consistently harmful on CEUS, showing that arbitrary perturbation is not a sufficient explanation. Photometric augmentation improved calibration and boundary behavior but did not improve overlap. The combined augmentation therefore appears to act as a balanced training distribution rather than as a single dominant trick. This interpretation aligns with domain-generalization literature showing that augmentation can improve robustness when it approximates plausible acquisition variation, but can degrade performance when it introduces unhelpful or excessive distortions (21–25).

The additional analyses conducted in this revision further strengthen and contextualize these findings. The BUS-to-CEUS domain-shift characterization (Section 3.6) confirmed that the two modalities differ not merely in brightness but in coordinated intensity, texture entropy, and high-frequency sharpness, providing an imaging-physical rationale for the three chosen perturbation families. Five-fold cross-validation (Section 3.7) showed that the augmentation effect was positive on average across data splits: the mean paired CEUS Dice difference was +0.009 ± 0.013 across folds, with 3 of 5 folds showing clear improvement, and the direction of effect was consistent with the single-split analysis. The multi-model comparison (Section 3.8) demonstrated that the augmentation benefit is not specific to the Residual U-Net backbone: all three architectures showed CEUS Dice improvement (range +0.008 to +0.048) and pixel ECE reduction, with all three architectures showing positive mean Dice differences. Finally, the TTA comparison (Section 3.9) showed that four-view TTA improved CEUS Dice but required 3.9-fold longer inference time, while training-only augmentation adds no inference overhead. However, these analyses also revealed that the improvement was modest in magnitude and should be interpreted as a methodologic signal rather than a definitive performance breakthrough. Taken together, the new experiments support the conclusion that the combined augmentation provides a positive but limited robustness benefit, and that the contribution lies in systematic evaluation rather than in a single high-performing configuration.

The comparison with architecture baselines is also informative. U-Net, Residual U-Net, and Attention U-Net had similar internal two-dimensional ultrasound Dice, but their CEUS behavior differed. The multi-model analysis (Section 3.8) further showed that the augmentation benefit was consistent across U-Net, Residual U-Net, and Attention U-Net, with all three architectures showing CEUS improvement. This does not argue against attention mechanisms in general; instead, it suggests that, for this dataset and task, a clinically motivated training distribution was at least as useful as adding a common architectural refinement. This distinction is important for medical AI manuscripts because a methodologic contribution should be tied to the clinical imaging problem. Here, CEUS appearance shift motivated the augmentation profile, and the efficiency analysis confirmed that the resulting method did not increase inference-time complexity.

The study should be interpreted within defined boundaries. The public package was image-level, so the analysis could not account for patient clustering, scanner manufacturer, center, acquisition protocol, histopathologic subtype, or reader variability, and patient-level baseline characteristics were not constructed. Critically, because patient identifiers were unavailable, patient-level separation across the development, internal test, and CEUS subsets could not be guaranteed; images from the same patient may appear in different subsets, which may inflate performance estimates and compromise the validity of statistical inference. The five-fold cross-validation added in this revision is likewise image-level and cannot resolve this patient-level leakage concern. The CEUS subset contained 170 images and was used as a CEUS domain-shift stress test rather than a patient-level validation cohort. The task was segmentation only, so no claim is made about benign-malignant diagnosis, surgical triage, outcome prediction, or replacement of O-RADS or IOTA-based assessment. CEUS HD95 remained persistently high (mean 199.7 pixels (baseline) and 197.6 pixels (combined); median ~187 pixels (IQR 154–237) on 512 × 512 images), indicating that boundary outliers remain a major limitation despite modest improvements in average overlap and calibration. HD95 was computed on resized 512 × 512 images and should not be interpreted as a physical millimeter error. The primary analysis used a deterministic fixed split and was supplemented by image-level five-fold cross-validation under the same predefined training configuration. Accordingly, the method should be viewed as a reproducible robustness strategy of limited magnitude that requires patient-level, multi-center, and reader-aware validation before clinical deployment (31–35).

Despite these boundaries, the work supports a methodologic direction for ovarian ultrasound AI. For limited public datasets and single-GPU research settings, carefully designed training-only augmentation can provide a small, balanced improvement in CEUS robustness and calibration without complicating inference. However, the gains are modest, and not all augmentation components were beneficial—noise augmentation was consistently harmful. Combined ultrasound-specific augmentation is therefore a useful but limited methodologic lever, not a solution to the BUS–CEUS domain shift. Future studies should combine this approach with prospective annotation review and diagnostic or measurement endpoints that connect segmentation quality to clinically interpretable outcomes, consistent with broader recommendations for moving medical AI from technical performance toward clinical impact (42). More broadly, prior work has shown that image-domain filtering choices can materially affect segmentation performance, reinforcing the need to evaluate preprocessing strategies empirically rather than assume that they are uniformly beneficial (43).

## 5 Conclusion

In this public image-level study of ovarian tumor ultrasound segmentation, a lightweight Residual U-Net trained with combined ultrasound-specific augmentation yielded a small but directionally favorable improvement in CEUS overlap, boundary behavior, and pixel calibration while preserving internal two-dimensional ultrasound performance. The benefit came from training-time distribution design rather than added inference-time model complexity. These findings support training-distribution design as a practical low-cost strategy for modality-shift robustness and warrant further patient-level and multi-center validation.

## Data Availability

Publicly available datasets were analyzed in this study. This data can be found at: https://doi.org/10.6084/m9.figshare.25058690.v2.

## Supplementary material

SUPPLEMENTARY FIGURE S1 BUS-to-CEUS domain-shift characterization. **(A)** Distribution of mean image intensity for B-mode ultrasound (BUS) and contrast-enhanced ultrasound (CEUS), showing CEUS images are systematically darker (BUS mean 58.5, CEUS mean 32.6). **(B)** Distribution of Shannon entropy, showing lower texture complexity in CEUS (BUS 6.53, CEUS 5.66). **(C)** Distribution of Laplacian sharpness variance, showing higher high-frequency content in CEUS (BUS 671, CEUS 857). **(D)** Box plots of dynamic range and lesion area fraction. These quantitative differences provide imaging context for the selected perturbation families.

SUPPLEMENTARY FIGURE S2 Model efficiency. **(A)** Parameter counts for selected models. **(B)** Inference time per image. **(C)** Peak GPU memory during fixed inference measurement.
