# Supplementary figures and images for: Training-only ultrasound-specific augmentation for ovarian tumor segmentation across B-mode and contrast-enhanced ultrasound
Source: Front Med (Lausanne). 2026 Jul 13;13:1878351. doi: 10.3389/fmed.2026.1878351 (PMC13402114; doi:10.3389/fmed.2026.1878351)

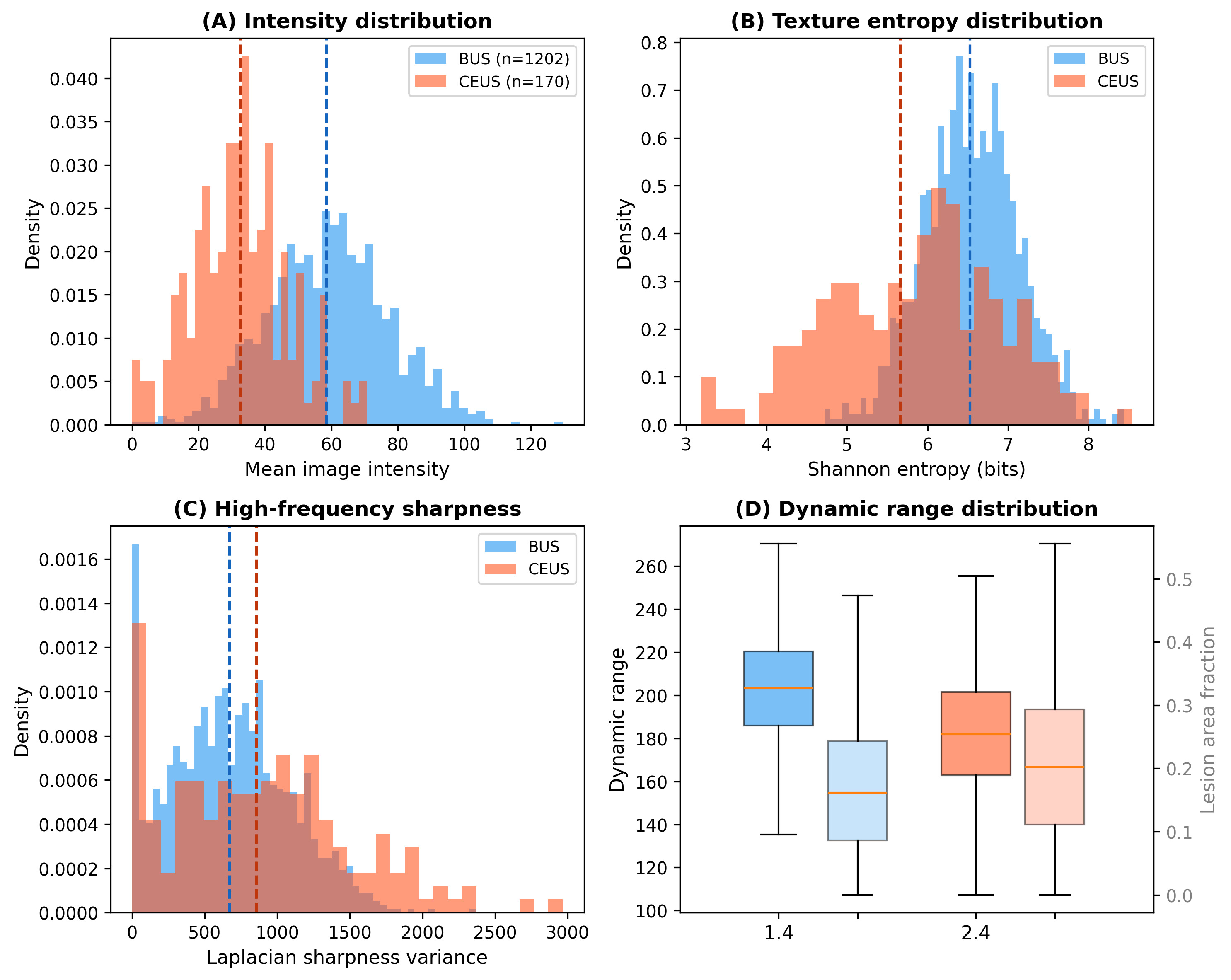

Supplement: SUPPLEMENTARY FIGURE S1 — BUS-to-CEUS domain-shift characterization. (A) Distribution of mean image intensity for B-mode ultrasound (BUS) and contrast-enhanced ultrasound (CEUS), showing CEUS images are systematically darker (BUS mean 58.5, CEUS mean 32.6). (B) Distribution of Shannon entropy, showing lower texture complexity in CEUS (BUS 6.53, CEUS 5.66). (C) Distribution of Laplacian sharpness variance, showing higher high-frequency content in CEUS (BUS 671, CEUS 857). (D) Box plots of dynamic range and lesion area fraction. These quantitative differences provide imaging context for the selected perturbation families. [file Image_1.JPEG]

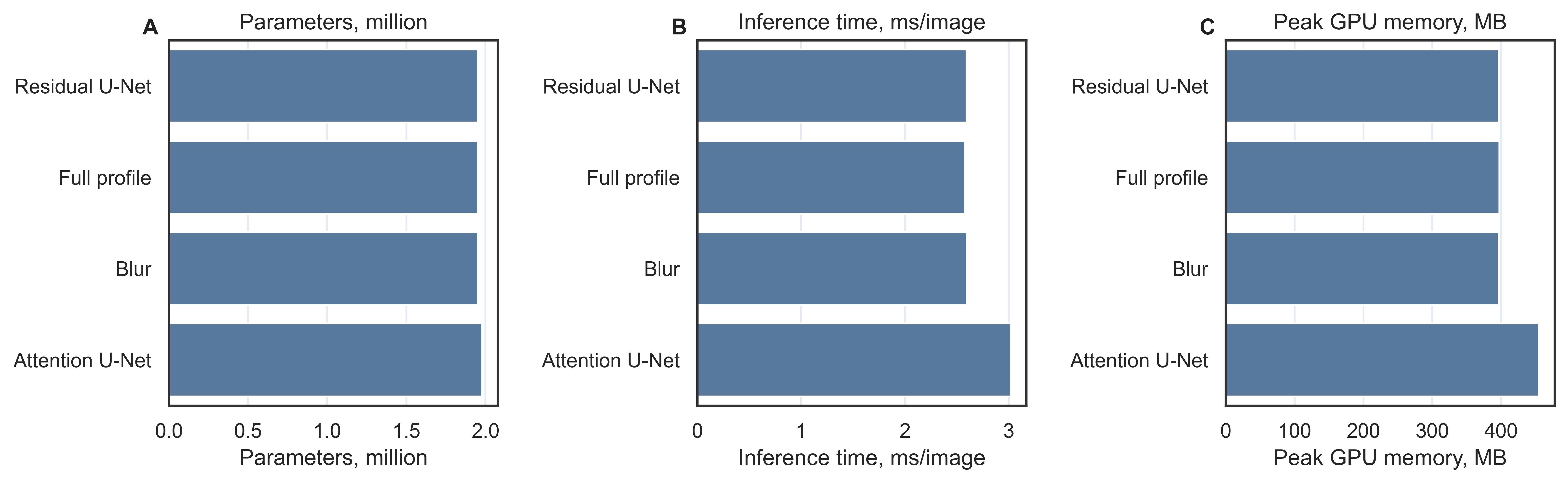

Supplement: SUPPLEMENTARY FIGURE S2 — Model efficiency. (A) Parameter counts for selected models. (B) Inference time per image. (C) Peak GPU memory during fixed inference measurement. [file Image_2.JPEG]
