# Supplementary material for: Training-only ultrasound-specific augmentation for ovarian tumor segmentation across B-mode and contrast-enhanced ultrasound
Source: Front Med (Lausanne). 2026 Jul 13;13:1878351. doi: 10.3389/fmed.2026.1878351 (PMC13402114; doi:10.3389/fmed.2026.1878351)
Supplement: Supplementary file 3 [file Table_8.DOCX]

**Supplementary Table S1.** Augmentation operations, parameters, and selection rationale.

| **Operation** | **Probability** | **Parameter range** | **Applied to** | **Selection rationale** |
| --- | --- | --- | --- | --- |
| Horizontal flip | 0.5 | — | Image and mask | Standard geometric augmentation |
| Vertical flip | 0.5 | — | Image and mask | Standard geometric augmentation |
| Brightness adjustment | 0.5 | Factor 0.85–1.15 | Image only | Simulate gain variation |
| Contrast adjustment | 0.5 | Factor 0.85–1.15 | Image only | Simulate gain variation |
| Gamma transformation | 0.5 | γ = 0.75–1.35 | Image only | Cover moderate brightness range from gain/mode differences |
| Gaussian blur | 0.35 | Radius 0.2–1.1 px | Image only | Approximate resolution loss from probe frequency differences |
| Multiplicative speckle noise | 0.55 | σ = 0.02–0.08 | Image only | Simulate ultrasound speckle variation |
| Additive Gaussian noise | 0.35 | σ = 0.005–0.025 | Image only | Simulate low-amplitude electronic/intensity noise |
